# Is representativeness the right question?

**DOI:** 10.1093/ije/dyt264

**Published:** 2014-01-15

**Authors:** C Mary Schooling, Heidi E Jones

**Affiliations:** City University of New York School of Public Health and Hunter College, New York, USA

We agree completely that representative studies are immensely valuable for describing disease patterns, quantifying the burden of disease^1^ and generating risk stratification models.^2^ Given representativeness is time- and place-specific,^3^ these all need regular updates and more representative studies. For example, the SCORE (Systematic COronary Risk Evaluation) system for predicting fatal cardiovascular disease (CVD) uses the same risk factors in different models for high- and low-CVD-risk European countries,^4^ but over time countries may, also, be promoted from high to low risk.^5^ Clearly, such risk prediction models are not scientific models that describe nature consistently across space and time,^6^ but they are immensely useful for service planning, targeting treatment and saving lives. Conversely, experimental studies, such as animal models and randomized controlled trials (RCTs), do not require representativeness to test scientific models.^6^

On the other hand, whether observational epidemiological studies, representative or not, are useful for generating hypotheses or testing causal factors in scientific models is less clear. First, these represent the triumph of hope over experience.^7^ Second, as was pointed out over 20 years ago, nearly all possible hypotheses have already been generated.^8^ Third, some potentially relevant hypotheses may not be readily observed for conceptual or practical reasons. The current paradigm may exclude some hypotheses as impossible, making them imperceptible. Apart from well-known biases inherent in observational studies, causal factors may be invariant in commonly studied populations, expensive or difficult to measure, affected by preclinical disease or hidden within the (mis)classification of diseases by symptom rather than cause. Fourth, as a discipline we have not generally thought through the hierarchy of studies to refute a hypothesis. Our current methods, using the Bradford-Hill viewpoints as a touchstone, are much more focused on corroborating hypotheses, with an RCT as the pinnacle of corroboration. However, even something as simple as ‘field’ epidemiology may refute hypotheses. For example, the existence of populations with low birthweight and low rates of heart disease casts doubt on a major role of birthweight in heart disease.^9^

Given these issues if we want to make progress in identifying causal processes in population health, assuming it is possible,^10^ rather than focusing on representativeness in studies used to generate or test (corroborate) hypotheses, it might be more useful to look for better ways to generate and screen plausible hypotheses, before we test them in suitable studies.^11^ Other methods of generating hypotheses about the drivers of population health are not obvious, but include using general mechanistic principles, starting with effective treatments and taking advantage of mechanistic insights from genetics or RCTs which include potential mediators. Not only do we need to move on from the debate about representativeness, we need to move onto some different questions.
